# Supplementary material for: Identification of Plasma Growth Factors and Cytokines as Diagnostic Biomarkers for the Lafora Form of Progressive Myoclonus Epilepsy
Source: Int J Mol Sci. 2025 Jun 3;26(11):5354. doi: 10.3390/ijms26115354 (PMC12155511; doi:10.3390/ijms26115354)
Supplement: Supplementary file 1 [file ijms-26-05354-s001.zip › ijms-3653934-supplementary.pdf]

Supplementary Material

Supplementary Figure S1: Cytokine levels elevated in mouse models of LD are not differentially present in the plasma of LD patients in comparison to healthy controls. Plasma levels of CXCL10 (A), S100B (B) and CCL20 (C) were analyzed in four samples from healthy subjects (pale blue) and seven samples from LD patients (pale pink) by using ELISA assays. Results are expressed as median with a range. Statistical differences between the groups were assessed using the Mann-Whitney non-parametric t-test. P-values are indicated.

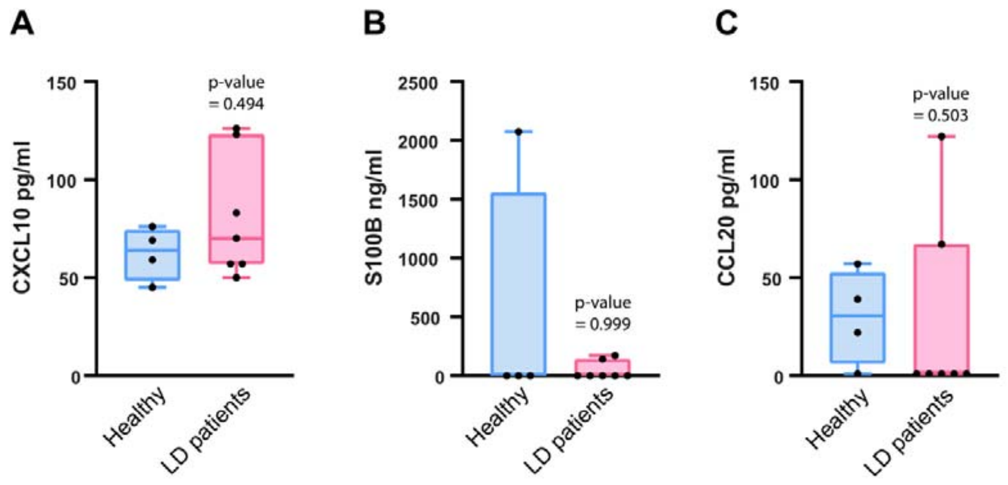

Supplementary Table S1. Description of the LD patients used in this work. The number of the patient, the affected gene and the mutation (both at DNA and protein level), gender, age at clinical onset and clinical presentation at different times of recruitment. The phenotytic stage of the patient according to Kim et al., 2021 is also indicated.

Lafora Patient ID	Gene	Mutation		SEX (M/F)	Age range at clinical onset (years)	First symptom	SEIZURES			Other seizures types at onset	Cognitive impairment at onset	Dementia at onset	Gait impairment at onset	Speech impairment at onset
		DNA	Protein				GTC seizures at onset	Myoclonic seizures at onset	Visual seizures at onset					
**LD1**	EPM2A	c.877C>T homozygous	p.Gln293Ter	F	6-10	Visual seizures	Yes	Yes	Yes	Atonic, Absences	Yes	Yes	Yes	Yes
**LD2**	EPM2A	c.508C>T homozygous	p.Pro170Ser	M	6-10	Visual seizures	Yes	Yes	Yes	no	Yes	Yes	Yes	Yes
**LD3**	EPM2B	c.205C>G homozygous	p.Pro69Ala	F	11-15	Visual and myoclonic seizures	Yes	Yes	Yes	absences, atonic	(diagnosed with ADHD)	Yes	Yes	Yes
**LD4**	EPM2A	c.721 C>T and exon 2 deletion	p.Arg241*	M	11-15	Cognitive	Yes	Yes	Yes	absences	Yes	no	no	no
**LD5**	EPM2A	c.163C>T / c.835G>A	p.Gln55X / p.Gly279Ser	M	21-25	GTCS	Yes	Yes	Yes	no	no	no	no	no
**LD6**	EPM2A	c.259A>G homozygous	p.Lys87Glu	F	asymptomatic	no	no	no	no	no	no	no	no	no
**LD7**	EPM2A	c.259A>G homozygous	p.Lys87Glu	M	1-5	Cognitive	no	no	no	no	Yes	no	no	Yes
**LD8**	EPM2B	c.348C>A homozygous	p.Cys116Ter	F	11-15	GTC and visual seizures	Yes	Yes	Yes	no	Yes	Yes	Yes	Yes
**LD9**	EPM2A	c.834C>A / c.108_139del	p.Cys278* / p.Ala37fs	M	11-15	GTC and visual seizures	Yes	Yes	Yes	absences	Yes	Yes	Yes	Yes
**LD10**	EPM2B	c.205C>G / c.1133T>C	p.Pro69Ala / p.Leu378Pro	F	11-15	GTCS	Yes	Yes	no	absences	no	no	no	no
**LD11**	EPM2B	c.205C>G / c.1133T>C	p.Pro69Ala / p.Leu378Pro	F	11-15	Myoclonic seizures	Yes	Yes	no	absences	Yes	no	no	no

Lafora Patient ID	T1						T2						T3					
	AGE range T1	CURRENT TREATMENT	GTCS FREQUENCY	MYOCLONIC SEIZURES FREQUENCY	OTHER SEIZURES FREQUENCY	Stage	AGE T2	CURRENT TREATMENT	GTCS FREQUENCY	MYOCLONIC SEIZURES FREQUENCY	OTHER SEIZURES FREQUENCY	Stage	AGE T3	CURRENT TREATMENT	GTCS FREQUENCY	MYOCLONIC SEIZURES FREQUENCY	OTHER SEIZURES FREQUENCY	Stage
**LD1**	16-20	VPA, CZP, PER, metformin	0	1/month	Visual: 1-3/month	**III**	16-20	VPA, CZP, PER, metformin	0	1/week-1/month	Visual: 1-2/month; Atonic: 1/2-3 months	**III**	16-20	VPA, CZP, PER, metformin	0	Daily, uncountable	Atonic: 1/week-1/month; Absences: 1/week-1/month	**IV**
**LD2**	16-20	VPA, PER, BRV, atkins diet	1/week	Daily, uncountable	Visual: 1/2-3 months	**IV**	sample not obtained						sample not obtained					
**LD3**	16-20	VPA, LEV, ZNS, CZP	1/month	Daily, uncountable	Visual: 1-2/month; Absences: daily and uncountable	**IV**	16-20	VPA, LEV, ZNS, CZP	1-7/month	Daily, uncountable	Visual: 0-3/week; Absences: daily and uncountable; Atonic: 1/month	**IV**	16-20	VPA, LEV, ZNS, CZP, ketogenic diet	5-11/month	Daily, uncountable	visual: 1/month; absences: daily and uncountable; Atonic: 5/6 months	**V**
**LD4**	16-20	VPA	0	1/2-3 month	Visual: 1/month	**II**	16-20	VPA, CBD, TPM, metformin	1/year	1/month	Visual: 1/month	**II**	sample not obtained					
**LD5**	31-35	VPA, CZP, metformin	0	0	no	**I**	31-35	VPA,CZP, metformin	0	0	no	**I**	sample not obtained					
**LD6**	6-10	no	no	no	no	**0**	6-10	no	no	no	no	**0**	6-10	no	no	no	no	**0**
**LD7**	11-15	no	no	no	no	**0**	11-15	no	no	no	no	**0**	11-15	no	no	no	no	**0**
**LD8**	16-20	VPA, LEV, CZP, ZNS, metformin	1-4/month	Daily, uncountable	no	**IV**	sample not obtained						sample not obtained					
**LD9**	11-15	ZNS, VPA, CLB, metformin	0-5/month	Daily, uncountable	no	**II**	sample not obtained						sample not obtained					
**LD10**	11-15	VPA, metformin	1/ year (she has had only 1 GTCS since clinical onset at that visit)	1/day-1/week	absences: 1/week	**I**	11-15	VPA, CZP, metformin	1/week - 1/month	Daily	no	**I**	sample not obtained					
**LD11**	11-15	VPA, CZP, metformin	1/ year (she has had only 1 GTCS since clinical onset at that visit)	Daily	no	**I**	11-15	VPA, CZP, BRV, metformin	1/week - 1/month	Daily, uncountable	no	**II**	sample not obtained					

M: male; F: female
BRV: brivaracetam; CBD: cannabidiol; CLB: clobazam; CZP: clonazepam; LEV: levetiracetam; PER: perampanel; TPM: topiramate; VPA: valproic acid; ZNS: zonisamid

GTCS: generalized tonic clonic seizures

ADHD: attention deficit hyperactivity disorder

Supplementary Table S2. Plasma-EDTA samples from LD patients and healthy controls were analyzed using the Proteome Profiler Human Cytokine Array kit (R&D systems). This array consists of 111 different captured antibodies spotted on a nitrocellulose membrane for the detection of multiple cytokines, chemokines, growth factors, and other soluble proteins. The intensity of the signals was analyzed using the Quick Spot software provided by the manufacturer. The analysis of the differential expression between healthy and LD samples was performed by using the FLASKI software (http://flaski.age.mpg.de). In this work, we focus our attention on mediators with a fold change between healthy and LD samples greater than 2 or less than 0.5 for further analysis. Average intensity of the spots determined by the Quick Spot software in the Proteome Profiler Human Cytokine Array Kits. Eight healthy and eleven samples from independent LD patients were analyzed as described in Materials and Methods. Fold change of the Healthy vs LD samples and the corresponding p-values, calculated by using an unpaired non-parametric t-test (Mann-Whitney test) is also indicated. In the Table we highlight in orange the mediators with a fold change <0.5 or >2, with a p-value lower than 0.05. Mediators are ordered according to fold change. Values corresponding to Cxcl10 and Ccl20 are highligted in grey.

**Healthy vs Patients**	**Average**<br>**Healthy (n=8)**	**Patients (n=11)**	**Healthy vs LD**<br>**Fold Change<0.5 or >2**	**p-value**
NC (Negative control)	0,00	0,00		
PDGF-AB/BB	9000,41	1065,69	8,45	0,0001
PDGF-AA	7197,94	1420,41	5,07	0,0009
EGF	2406,53	480,82	5,01	0,0008
ENA-78/CXCL5	2292,20	720,45	3,18	0,0218
TARC/CCL17	1128,13	399,52	2,82	0,0180
BDNF	1792,01	642,62	2,79	0,0011
MIF	4376,88	1787,67	2,45	0,0002
Thrombospondin-1	5575,32	2360,47	2,36	0,0090
Angiopoietin-1	1850,48	821,64	2,25	0,0146
RANTES/CCL5	14307,91	6510,20	2,20	0,0707
Dkk-1/Dickkopf-1	1268,62	584,47	2,17	0,0157
Growth Hormone	2559,68	1238,98	2,07	0,4164
Serpin E1	15889,21	8103,80	2,00	0,0063
CD31	18348,39	12031,88	1,52	0,1199
IL-2	391,90	267,47	1,47	0,2843
IL-10	357,41	256,53	1,39	0,3196
IL-3	323,89	232,67	1,39	0,3555
TGF-alpha	264,78	191,22	1,38	0,4326
EMMPRIN	8300,32	6101,65	1,36	0,1364
VEGF	420,22	314,19	1,34	0,2412
I-TAC	275,11	217,39	1,27	0,3339
PF4	21262,51	16987,93	1,25	0,2545
MCP-1	698,33	558,68	1,25	0,2874
Fas Ligand	686,93	554,64	1,24	0,5538
IL-1beta	111,63	91,02	1,23	0,7483
GM-CSF	436,19	360,91	1,21	0,3430
Vitamin D BP	10864,33	9102,84	1,19	0,4316
Lipocalin-2	9882,83	8477,77	1,17	0,4670
FGF basic	992,51	853,65	1,16	0,4216
IL-11	688,08	598,57	1,15	0,4238
FGF-7	174,98	153,65	1,14	0,7732
Cystatin C	6093,82	5390,25	1,13	0,5752

MCP-3	154,34	139,17	1,11	0,8269
TFF3	7729,50	6988,91	1,11	0,5960
IL-13	66,18	59,91	1,10	0,8755
IL-22	516,29	468,56	1,10	0,6680
IL-27	281,25	256,19	1,10	0,8060
Cripto-1	299,85	277,41	1,08	0,7528
IL-32	219,52	204,49	1,07	0,8368
Flt-3 Ligand	244,06	227,41	1,07	0,7906
GRO-alpha	238,85	222,56	1,07	0,8229
MMP-9	15859,98	14827,37	1,07	0,7042
TNF-alpha	652,80	616,97	1,06	0,8527
G-CSF	195,18	186,68	1,05	0,9013
Angiopoietin-2	902,95	864,41	1,04	0,8443
CD40 ligand	2434,73	2330,87	1,04	0,8003
SHBG	10524,95	10123,08	1,04	0,8137
IL-17A	2350,17	2261,80	1,04	0,8087
IL-4	462,03	452,13	1,02	0,9034
TfR	3972,84	3948,10	1,01	0,9460
M-CSF	308,06	307,14	1,00	0,9880
IL-6	398,52	399,57	1,00	0,9902
C-Reactive Protein	24060,17	24255,02	0,99	0,9523
IGFBP-2	7508,16	7700,11	0,98	0,8607
RAGE	755,71	777,83	0,97	0,9135
IL-34	80,09	82,80	0,97	0,9415
HGF	332,92	344,46	0,97	0,8954
IL-19	389,85	405,23	0,96	0,8538
Complement Component C5/C5a	7221,63	7523,88	0,96	0,7021
IL-1ra	253,18	267,50	0,95	0,8693
SDF-1alpha	2842,56	3006,60	0,95	0,6945
Reference Spots	19042,48	20161,79	0,94	0,2931
FGF-19	1843,56	1968,06	0,94	0,7462
ST2	2140,07	2295,75	0,93	0,7729
Kallikrein 3	1082,75	1185,68	0,91	0,6796
IL-31	129,15	141,44	0,91	0,8449
RBP-4	25704,58	28187,33	0,91	0,2505
Chitinase 3-like 1	8507,89	9348,44	0,91	0,5968
Myeloperoxidase	514,78	565,93	0,91	0,6994
MIG	189,08	209,09	0,90	0,7377
MIP-1alpha/MIP-1beta	103,79	114,90	0,90	0,8182
IFN-gamma	496,76	550,25	0,90	0,7270
IGFBP-3	10917,62	12109,98	0,90	0,2035
IP-10/Cxcl10	500,24	557,10	0,90	0,8025
Apolipoprotein A-I	13499,03	15043,87	0,90	0,1510
Osteopontin	10506,06	11736,96	0,90	0,5789
Adiponectin	31728,76	35476,32	0,89	0,3049
VCAM-1	15482,02	17517,03	0,88	0,1261
ICAM-1	2569,58	2907,73	0,88	0,3953
Reference Spots	17661,36	20034,80	0,88	0,0936
Reference Spots	17588,28	20031,55	0,88	0,1631
IL-1alpha	556,35	633,69	0,88	0,5997
IL-12 p70	222,71	255,48	0,87	0,6310

IL-16	140,62	161,38	0,87	0,6350
IL-24	152,79	176,11	0,87	0,7165
Angiogenin	25792,29	29859,86	0,86	0,1652
Pentraxin-3	1360,08	1577,15	0,86	0,4438
CD14	2957,66	3447,92	0,86	0,2352
Resistin	2242,92	2630,73	0,85	0,4640
uPAR	1039,76	1257,79	0,83	0,2304
Complement Factor D	5417,08	6555,63	0,83	0,1382
Relaxin-2	324,35	395,80	0,82	0,2573
IL-5	109,84	136,05	0,81	0,6881
IL-18 BPa	9468,18	11763,38	0,80	0,0453
IL-15	52,10	64,95	0,80	0,6311
IL-23	212,67	270,58	0,79	0,4648
CD30	532,04	684,94	0,78	0,2812
MIP-3alpha/Ccl20	199,75	258,50	0,77	0,2040
IL-33	56,02	72,84	0,77	0,5874
Leptin (antes sí)	6051,72	7941,01	0,76	0,6168
LIF	118,66	158,21	0,75	0,5503
IL-8	402,26	538,16	0,75	0,2277
Endoglin	8078,73	10960,22	0,74	0,0557
BAFF	3210,84	4392,92	0,73	0,0970
DPPIV	12368,52	17106,04	0,72	0,0768
TIM-3/HAVCR2	4317,44	6438,51	0,67	0,0330
MIP-3beta	332,53	523,50	0,64	0,1826
GDF-15/MIC-1	1795,33	3690,24	0,49	0,0052

Supplementary Table S3. Values of EGF, PGDF-BB, BDNF, and MIF determined by ELISA assays in LD samples at different recruitment times (T1, T2 and T3), in healthy samples and in other monogenic epileptic patients. F, female; M, male; nd, not determined; ns, no sample obtained.

Lafora Patients	Age range (T1/T2/T3)	Sex	Gene	T1/T2/T3			
				EGF (pg/ml)	PDGF-BB (pg/ml)	BDNF (ng/ml)	MIF (ng/ml)
**LD1**	16-20	F	EPM2A	3/1/49	215/83/547	65/33/18	17/36/27
**LD2**	16-20	M	EPM2A	11/ns/ns	531/ns/ns	263/ns/ns	24/ns/ns
**LD3**	16-20	F	EPM2B	3/1/6	1/32/262	165/78/85	32/33/47
**LD4**	16-20	M	EPM2A	2/1/ns	273/784/ns	22/61/ns	24/36/ns
**LD5**	31-35	M	EPM2A	9/24/ns	85/1424/ns	49/73/ns	26/32/ns
**LD6**	6-10	F	EPM2A	6/58/2	8/1/64	28/24/95	26/34/43
**LD7**	11-15	M	EPM2A	1/1/31	476/140/1953	51/26/136	36/38/37
**LD8**	16-20	F	EPM2B	1/ns/ns	15/ns/ns	44/ns/ns	38/ns/ns
**LD9**	11-15	M	EPM2A	61/ns/ns	29/ns/ns	1/ns/ns	32/ns/ns
**LD10**	11-15	F	EPM2B	7/7/ns	1848/600/ns	137/31/ns	15/34/ns
**LD11**	11-15	F	EPM2B	2/3/ns	411/229/ns	14/29/ns	16/53/ns
**Healthy Controls**							
**H1**	26-30	F		210	1500	114	nd
**H2**	31-35	M		151	1433	141	nd
**H3**	21-25	F		180	1488	178	nd
**H4**	31-35	M		200	1277	125	nd
**H5**	21-25	F		221	12037	199	743
**H6**	26-30	M		nd	nd	nd	nd
**H7**	21-25	F		nd	nd	nd	nd
**H8**	21-25	M		237	16192	150	283
**H9**	21-25	F		230	8151	129	nd
**H10**	26-30	F		117	1681	170	370
**H11**	26-30	M		226	3734	201	565
**H12**	21-25	F		101	2424	83	375
**H13**	26-30	M		232	5004	75	nd
**Other monogenic epileptic patients**							
**OME1**	16-20	M	(SCNA1)	16	915	23	47
**OME2**	16-20	M	(SCNA1)	32	1067	29	39
**OME3**	26-30	M	(SCNA1)	38	2702	39	74
**OME4**	26-30	M	(SLC2A1)	101	2439	41	107
**OME5**	26-30	M	(SLC2A1)	160	3334	55	135
**Median LD values (T1/T2/T3)**				**03/ 03/ 18**	**215/ 185/ 405**	**49/ 32/ 88**	**26/ 35/ 40**
**Median control values**				**210**	**2424**	**141**	**375**
**Median Dravet values**				**32**	**1067**	**29**	**47**
**Median GLUT1 values**				**130**	**2887**	**48**	**121**
